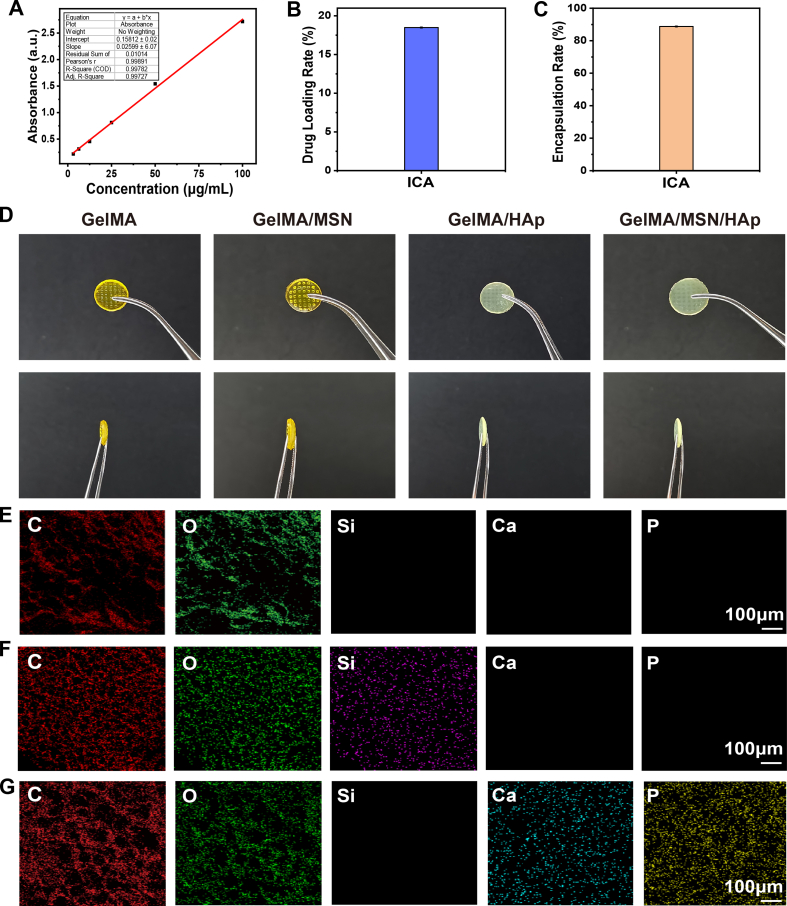# Lymphatic incorporated biomimetic scaffold enhances Osteoangio-lymphogenic coupling via HIF-1α mediated mitochondrial reprogramming for osteoporotic bone repair

**DOI:** 10.1016/j.bioactmat.2025.10.041

**Published:** 2025-11-04

**Authors:** Weipeng Sun, Qing Lin, Biyi Zhao, Minying Li, Jiacong Xiao, Xueshan Jin, Jinfu Liu, Yifei Wang, Ronghua Zhang, Xiaoyun Li, Ziwei Jiang

**Affiliations:** a State Key Laboratory of Bioactive Molecules and Druggability Assessment, Guangdong Basic Research Center of Excellence for Natural Bioactive Molecules and Discovery of Innovative Drugs, College of Traditional Chinese Medicine, Jinan University, No.601, West Huangpu Avenue, Tianhe District, Guangzhou, 510405, China; b The First Affiliated Hospital of Jinan University, No.613, West Huangpu Avenue, Tianhe District, Guangzhou, 510405, China; c School of Basic Medical Sciences, Guangzhou University of Chinese Medicine, No. 232, West Waihuan Road, Panyu District, Guangzhou Guangdong Province, 510006, China; d First Clinical College, Guangzhou University of Chinese Medicine, No. 12, Ji Chang Road, Baiyun District, Guangzhou, 510000, Guangdong Province, China; e Acupuncture and Rehabilitation Clinical Medicine Institute, Guangzhou University of Chinese Medicine, No. 232, West Waihuan Road, Panyu District, Guangzhou, 510006, Guangdong Province, China; f College of Pharmacy, Jinan University, Guangzhou, Guangdong Province, China; g Guangdong Provincial Key Laboratory of Traditional Chinese Medicine Informatization, Guangzhou, Guangdong Province, China; h College of Cancer Institute, Jinan University, Guangzhou University of Chinese Medicine, Guangzhou, Guangdong Province, China; i Department of Orthopaedics, The First Affiliated Hospital of Guangzhou University of Chinese Medicine, No. 12, Ji Chang Road, Baiyun District, Guangzhou, 510000, China

**Keywords:** Composite scaffold, Bone repair, Osteogenesis, Angiogenesis, Lymphangiogenesis

## Abstract

Recent studies have challenged the notion that bone tissue lacks lymphatic vessels, highlighting their essential role in bone regeneration. Effective bone repair relies on the interplay of osteogenesis, angiogenesis, and lymphangiogenesis. Here, we present a three dimensional-printed biomimetic Gelatin methacryloyl/icariin@Mesoporous silica nanoparticle/Hydroxyapatite (GelMA/ICA@MSN/HAp) scaffold designed to recruit bone marrow mesenchymal stem cells (BMSCs), human umbilical vein endothelial cells (HUVECs), and lymphatic endothelial cells (LECs), enabling these cells to self-assemble into “cell islands” that coordinate tissue regeneration. The scaffold exhibits excellent biocompatibility, biodegradability, and sustained cytokine release. In vitro, it promotes the migration, proliferation, and lineage-specific differentiation of BMSCs, HUVECs, and LECs. In an osteoporotic bone defect model, the scaffold significantly enhances new bone formation, supports vascular remodeling by recruiting HUVECs, and induces lymphatic maturation via LECs, accompanied by upregulation of bone morphogenetic protein 2, vascular endothelial growth factor, and prospero homeobox protein 1. Transcriptomic analysis identifies activation of the hypoxia-inducible factor 1 alpha (HIF-1α) signaling pathway as crucial to these effects. Mechanistically, LECs-derived conditioned medium stimulates HUVECs angiogenesis and BMSCs osteogenesis by inducing HIF-1α mediated mitochondrial metabolic reprogramming. This is the first study to integrate lymphatic modulation into scaffold design for bone repair, achieving osteo-angio-lymphogenic coupling and establishing a novel organoid model for bone regeneration.

## Highlights

• A 3D-printed GelMA/ICA@MSN/HAp scaffold recruits BMSCs, HUVECs, and LECs to form self-organized multicellular “cell islands” for bone repair.
• The scaffold integrates osteo–angio–lymphogenic coupling in one platform, driving robust and spatially organized bone regeneration.
• LECs-derived medium enhances angiogenesis and osteogenesis via HIF-1α–mediated mitochondrial metabolic reprogramming in HUVECs and BMSCs.
• This work presents the first scaffold-based strategy integrating lymphatic regulation and an organoid-like system for bone repair.

## Graphical abstract

(A) Schematic diagram of the preparation process for the GelMA/ICA@MSN/HAp scaffold. (B) Schematic diagram of the in vivo action mechanism of the GelMA/ICA@MSN/HAp scaffold. (C) HIF-1α mediated mitochondrial metabolic reprogramming promotes bone repair.

**Figure:**
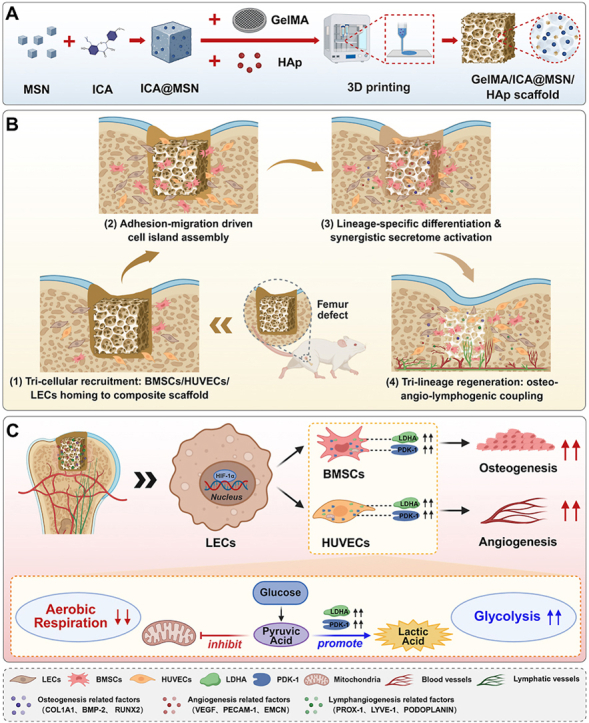

## 1 Introduction

Osteoporotic bone defects are a significant clinical challenge, severely affecting patients’ quality of life and imposing a substantial burden on healthcare systems [1,2]. Bone regeneration is a highly complex process regulated by the dynamic interplay between bone tissue, the vascular system and the lymphatic network [3,4]. Vascular endothelial cells promote osteogenesis through paracrine signaling, direct cell cell interactions and microenvironmental modulation [5]. In contrast, the role of the lymphatic system in bone regeneration has only recently been recognized. It was long believed that tissues like bone lacked lymphatic vessels. However, recent studies have definitively demonstrated their presence and critical function in bone regeneration [[6], [7], [8], [9]]. underscoring the importance of vascular and lymphatic balance in bone repair, particularly in large osteoporotic defects. This suggests that lymphatic modulation could offer a novel therapeutic strategy for enhancing bone formation.

Despite advances in surgical treatments, such as autologous and allogeneic bone grafts and artificial bone substitutes, these methods face limitations including immune rejection, pathogen transmission and suboptimal osseointegration [[10], [11], [12], [13], [14], [15], [16]]. Recently, nanomedicine based immunotherapy, organoid on chip models, and multifunctional biological scaffolds have attracted significant attention. However, they still suffer from critical drawbacks such as insufficient vascularization, adverse responses and inadequate osteogenic potential, which hinder their clinical translation [[17], [18], [19]]. Therefore, it is necessary to design and construct a strategy that provides sufficient osteogenesis and vascularization with fewer adverse effects.

Gelatin methacryloyl (GelMA) is a biomimetic extracellular matrix, providing both mechanical support and biochemical cues to promote bone tissue regeneration while effectively facilitating blood vessel and lymphatic vessel formation [[20], [21], [22], [23]]. Mesoporous silica nanoparticle (MSN) offers a large specific surface area and tunable pore size, making them suitable for efficient loading and sustained release of bioactive compounds such as icariin (ICA). They also help regulate scaffold porosity regulation and enhance cell adhesion [24]. Hydroxyapatite (HAp), the main inorganic component of natural bone, exhibits excellent osteoconductivity and mechanical reinforcement, fulfilling the load-bearing requirements of bone defect repair [[25], [26], [27]]. Moreover, the controlled release of calcium and phosphate ions from HAp stimulates osteoblast differentiation and accelerates bone matrix mineralization [28,29]. ICA, a major bioactive component derived from *Epimedium*, has demonstrated significant osteogenic potential in osteoporotic fracture models, with clinical studies supporting its efficacy in bone regeneration [[30], [31], [32], [33]]. When incorporated into biomaterials, ICA exhibits excellent biocompatibility and bone repair capabilities [34]. The combination of ICA with GelMA and HAp-based scaffolds presents a promising approach to coordinating osteogenesis, angiogenesis and lymphangiogenesis, ultimately enhancing bone regeneration.

To overcome current challenges in bone defect repair, this study introduces a paradigm shift by emphasizing the previously overlooked yet critical role of lymphatics in bone regeneration. We propose an innovative “osteo-angio- lymphogenic triple coupling” strategy and develop a three dimensional (3D) -printed biomimetic scaffold, GelMA/ICA@MSN/HAp, designed to synchronously promote osteogenesis, angiogenesis, and lymphangiogenesis within a single construct **(Graphical Abstract)**. Beyond conventional approaches, this scaffold not only recruits BMSCs, HUVECs, and LECs to form self-organized “cell islands” that spatially integrate multicellular functions, but also unveils a previously unrecognized mechanism: scaffold-activated LECs drive mitochondrial metabolic reprogramming in BMSCs and HUVECs via hypoxia-inducible factor 1 (HIF-1α) mediated paracrine signaling. Through comprehensive material characterization, in vitro bioactivity assays, and in vivo validation, we demonstrate that the scaffold significantly enhances bone regeneration. Furthermore, transcriptomic and mechanistic analyses identify the HIF-1α pathway as central to this process, with LECs derived CXCL12 contributing to the metabolic reprogramming essential for osteogenic and angiogenic differentiation. These findings not only establish our composite scaffold as a promising organoid-inspired platform for bone tissue engineering, but also reposition the lymphatic system as an active therapeutic target, opening new avenues for lympho-instructive biomaterial design.

## 2 Results

### 2.1 Characterization of ICA@MSN and HAp nanoparticle

We first loaded MSN with ICA to form ICA@MSN and observed its characteristics (Fig. 1A). The average particle size of MSN was 255 nm, which increased to 396 nm after loading with ICA (Fig. 1B and C). The Zeta potential measurements revealed that MSN had a potential of 4.60 ± 0.38 mV. Upon loading with ICA, the Zeta potential of significantly increased to 7.52 ± 0.77 mV (Fig. 1D). TEM images revealed that the morphology of HAp nanoparticles exhibited a rod-like structure, whereas MSN nanoparticles displayed a smooth, mesoporous spherical structure. Following ICA loading, the ICA@MSN particles retained their spherical morphology but showed a slight increase in size and a rougher surface texture, confirming the attachment of ICA molecules (Fig. 1E). The X-ray diffraction (XRD) analysis of HAp nanoparticles showed characteristic diffraction peaks at 26.0°, 31.9°, 40.0°, 46.8°, 49.6°, and 53.3° (Fig. 1F), indicating a well-defined crystalline structure with high crystallinity. In contrast, the XRD patterns of MSN and ICA@MSN displayed a broad diffraction peak at 22.9° (Fig. 1G). After loading with ICA, sharp diffraction peaks at 6.9°, 8.2°, 9.1°, and 21.8° emerged, indicating that an enhancement in crystalline. This suggested that ICA molecules, which likely possess an inherent crystalline structure, may have undergone partial crystallization upon interaction with the MS surface, further confirming the successful loading of ICA onto MSN. A characteristic ultraviolet visible spectroscopy (UV–Vis) absorption peak at 223 nm was observed for MSN, while a new absorption peak near 270 nm appeared in ICA@MSN, indicating the presence of ICA (Fig. 1H). To quantify the ICA content, a standard curve was constructed using ethanol solutions with ICA concentrations of 100, 50, 25, 12.5, 6.25, and 3.125 μg/mL, measuring absorbance at 270 nm. The resulting standard curve equation was y = 0.02599x + 0.15812 (Fig. S1A). Based on the absorbance of the supernatant after ICA loading, the drug loading capacity of ICA was calculated to be 18.49 ± 0.07 % (Fig. S1B), with an encapsulation efficiency of 88.77 ± 0.32 % (i.e., each milligram of MSN contains 0.173 mg of ICA) (Fig. S1C), highlighting the high efficiency of MSN as a carrier for ICA delivery.

**Fig. 1:**
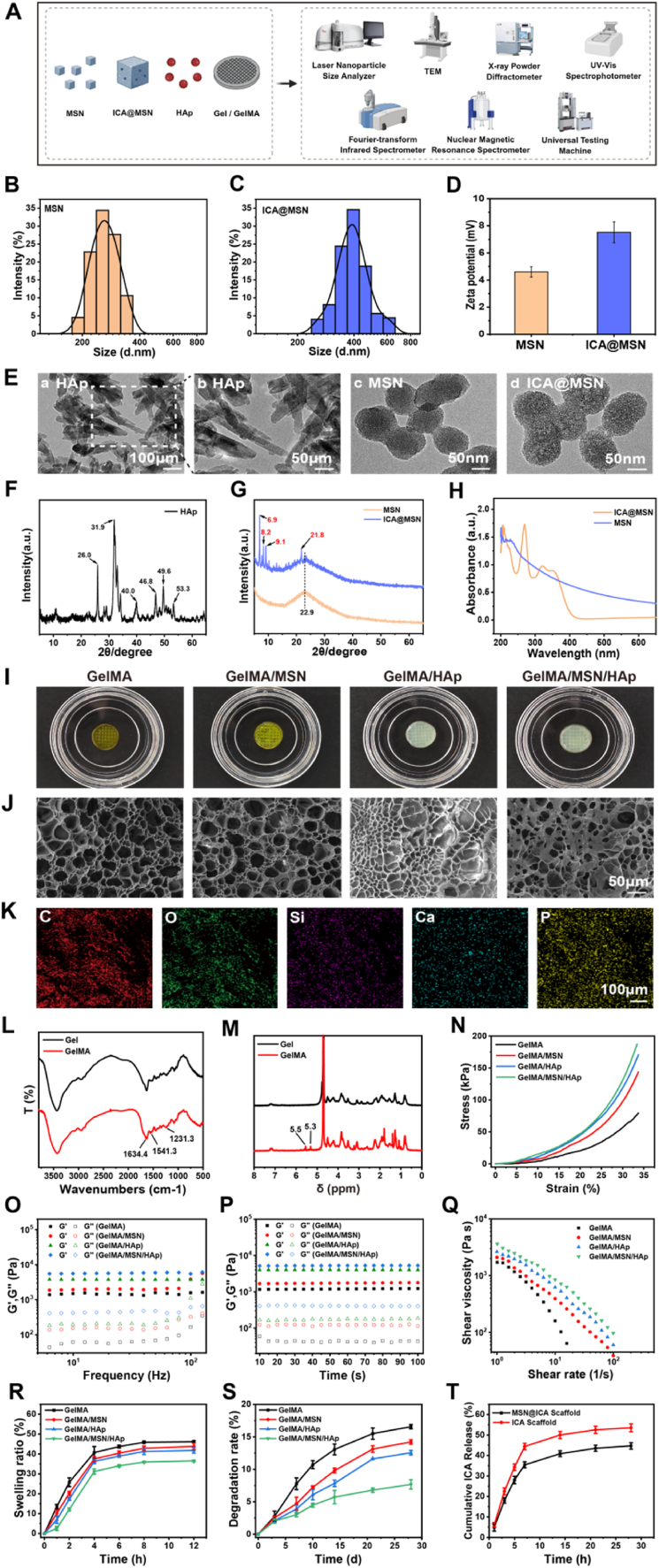
**Characterization of ICA@MSN, HAp nanoparticles and hydrogel materials.** (A) Schematic diagram illustrating the experimental design. (B, C) Particle size distribution plots of MSN and ICA@MSN. (D) Zeta potential plot of MSN and ICA@MSN. (E) TEM images of HAp, MSN and ICA@MSN: a, b. Rod-like structure of HAp nanoparticles; c. Mesoporous spherical structure of MSN nanoparticles; d. Rough surface morphology of ICA@MSN nanoparticles. (F) XRD diffraction pattern of HAp. (G) XRD diffraction patterns of MS and ICA@MSN. (H) UV–Vis absorption spectra of MS and ICA@MSN. (I) Front view of the hydrogel material's macroscopic appearance. (J) SEM images of the hydrogel material, scale bar = 50 μm. (K) Elemental analysis of the GelMA/MSN/HAp hydrogel material, scale bar = 100 μm. (L) FT-IR spectra of Gel and GelMA. (M) NMR spectra of Gel and GelMA. (N) Stress-strain curves of hydrogels with MSN and HAp. (O) Storage modulus (G′) and loss modulus (G″) as a function of frequency. (P) Storage modulus (G′) and loss modulus (G″) over time. (Q) Effect of MSN and HAp on the viscosity. (R) Swelling ratio of hydrogels with MSN and HAp. (S) Degradation rate of the hydrogels with MSN and HAp. (T) ICA release profile in GelMA hydrogel. Each experiment was performed at least three times, one-way ANOVA with Tukey's multiple comparisons.

### 2.2 Characterization of GelMA/ICA@MSN/HAp composite scaffold

Under white light, the hydrogel scaffold exhibited a well-defined, interconnected mesh with channel-like architecture (Fig. 1I). Scanning electron microscope (SEM) and elemental analysis confirmed a hierarchical porous structure micropores within macropores facilitating cell infiltration, adhesion, and proliferation. Energy dispersive X-ray spectroscopy (EDS) mapping showed uniform distribution of Si, Ca, and P, verifying successful incorporation of MSN and HAp into the GelMA matrix (Fig. 1J and K). At the same time, Fourier transform infrared spectroscopy (FT-IR) analysis showed enhanced amide I (1634.4 cm^−1^), II (1541.3 cm^−1^), and III (1231.3 cm^−1^) peaks in GelMA *vs*. Gel (Fig. 1L), indicating the introduction of methacrylate groups. NMR detected acrylic protons at 5.3 and 5.5 ppm, confirming GelMA synthesis (Fig. 1M). Mechanical testing (Fig. 1N) showed increased compressive strength with MSN and HAp, likely due to enhanced crosslinking and intermolecular interactions. Rheology (Fig. 1O and P) demonstrated that storage modulus (G′) consistently exceeded loss modulus (G″), confirming stable gel formation. G′ and G″ values were higher with MSN and HAp, indicating improved elasticity and viscoelasticity. Viscosity analysis revealed the highest viscosity in the GelMA/MSN/HAp group, attributed to hydrogen bonding with MSN and restricted chain mobility from HAp (Fig. 1Q). Swelling behavior showed reduced swelling with MSN and HAp due to higher crosslinking and physical water barriers (Fig. 1R). Equilibrium swelling was reached after 8 h, suggesting improved structural stability for implantation. Degradation analysis showed slower degradation with MSN and HAp, especially in GelMA/MSN/HAp (Fig. 1S), ensuring prolonged structural support during bone regeneration. Drug release studies showed a biphasic release of ICA: ∼35 % released in the first 7 days, ∼45 % by day 28. MSN enabled sustained delivery, supporting therapeutic efficacy for bone repair (Fig. 1T).

### 2.3 The GelMA/ICA@MSN/HAp composite scaffold promotes recruitment and osteogenic differentiation of BMSCs

**Fig. 2:**
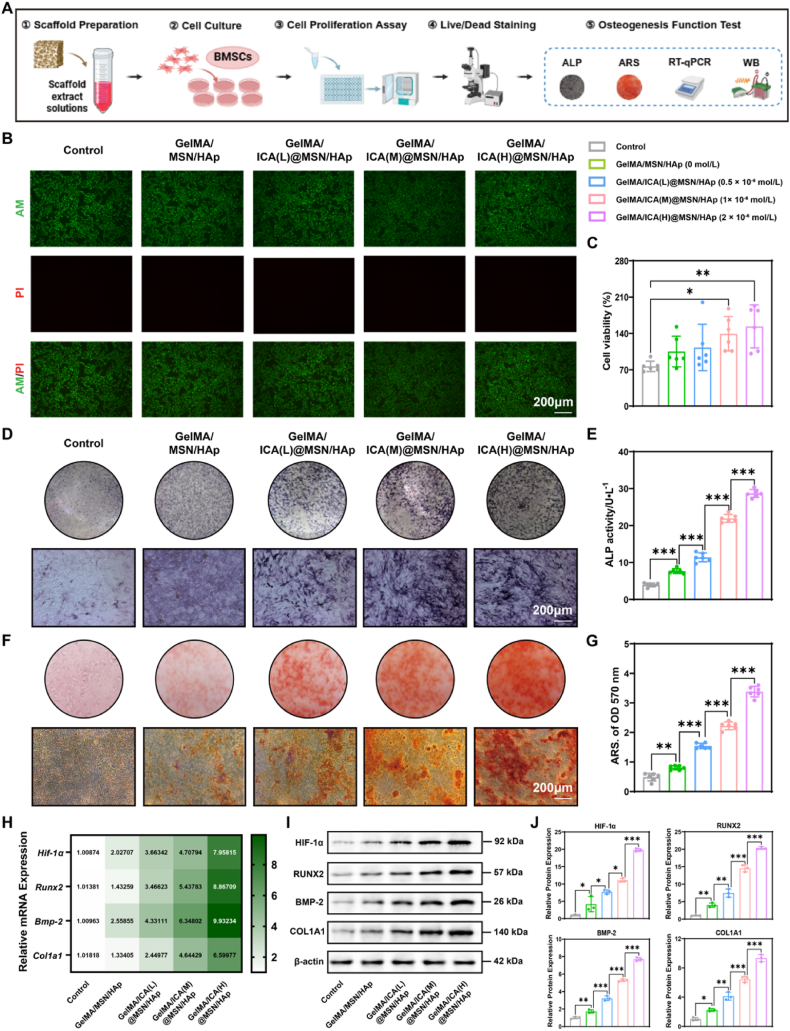
**GelMA/ICA@MSN/HAp composite scaffold promotes BMSCs proliferation and osteogenic differentiation.** (A) Schematic diagram illustrating the experimental design. (B) Inverted fluorescence microscope images of live/dead cells double fluorescent staining, scale bar = 200 μm. (C) Cell proliferation activity of BMSCs in different groups. (D–E) ALP staining and quantitative analysis, scale bar = 200 μm. (F–G) ARS staining and quantitative analysis, scale bar = 200 μm. (H) Relative expression of osteogenic differentiation related mRNA in BMSCs (*Hif-1α, Runx2, Bmp-2, Col1a1*). (I–J) Relative expression of osteogenic differentiation related proteins in BMSCs (HIF-1α, RUNX2, BMP-2, COL1A1), normalized to β-actin. ∗*P* < 0.05, ∗∗*P* < 0.01, ∗∗∗*P* < 0.001, each experiment was performed at least three times, one-way ANOVA with Tukey's multiple comparisons.

We systematically evaluated the biocompatibility and osteogenic potential of the GelMA/ICA@MSN/HAp composite scaffold using a comprehensive experiment approach (Fig. 2A). GelMA/ICA@MSN/HAp composite scaffold significantly enhanced BMSCs proliferation activity, indicating excellent cytocompatibility. Notably, the high-concentration ICA scaffold group showed the most rapid cell proliferation after 24 h (Fig. 2C). Live/dead staining further confirmed high cell viability and density within the scaffold, with BMSCs displaying spindle-shaped morphology and extended pseudopodia - indicative of active cell - scaffold interactions and effective cell recruitment (Fig. 2B). Furthermore, the high-concentration ICA group exhibited markedly higher alkaline phosphatase (ALP) activity and a greater alizarin red S (ARS) stained mineralization area compared to the control group (Fig. 2D–G), indicating enhanced osteogenic potential. At the same time, Western blotting revealed a dose-dependent upregulation of osteogenic differentiation related proteins, including runt related transcription factor 2 (RUNX2), collagen type Ⅰ alpha 1 (COL1A1) and bone morphogenetic protein 2 (BMP-2). These findings were further supported by Real-Time quantitative polymerase chain reaction (RT-qPCR) analysis, which showed consistent increases in corresponding mRNA levels (Fig. 2H, I, J). Collectively, these results demonstrate that the GelMA/ICA@MSN/HAp scaffold promotes both the proliferation and osteogenic differentiation of BMSCs in a concentration-dependent manner.

### **2.4** **The GelMA/ICA@MSN/HAp composite scaffold promotes recruitment and angiogenic differentiation of** HUVECs

**Fig. 3:**
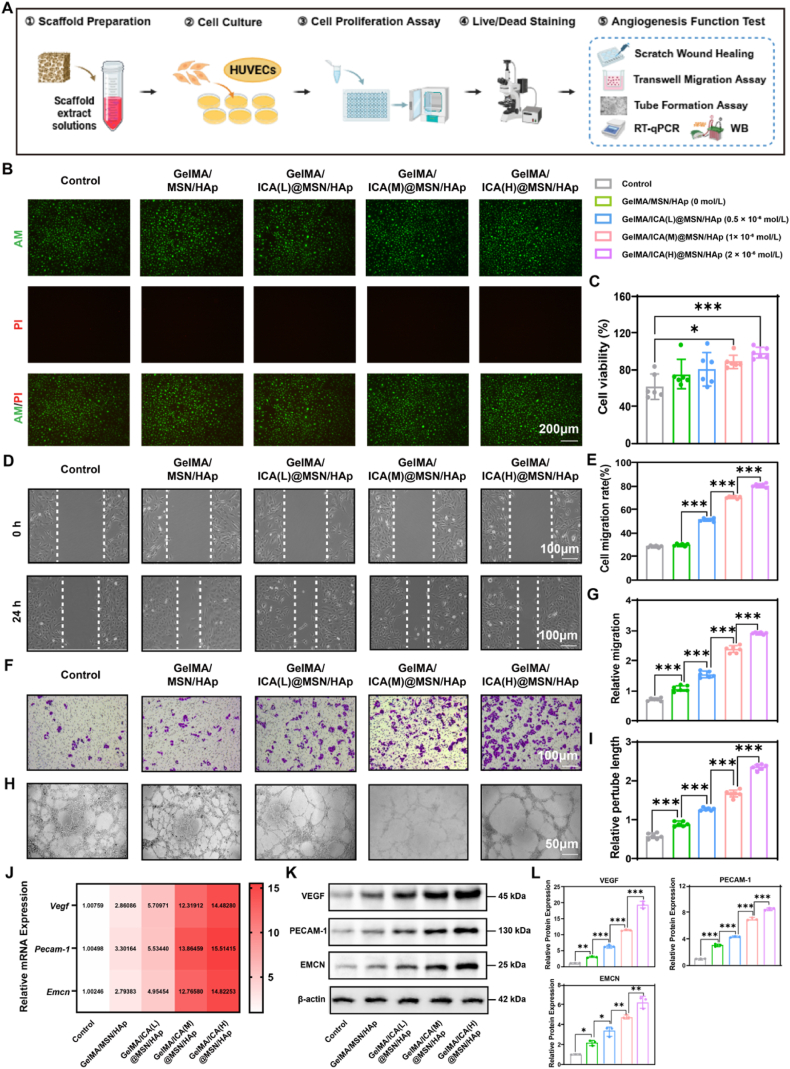
**GelMA/ICA@MSN/HAp composite scaffold promotes HUVECs proliferation and angiogenesis differentiation.** (A) Schematic diagram illustrating the experimental design. (B) Inverted fluorescence microscope images of live/dead cells double fluorescent staining, scale bar = 200 μm. (C) Cell proliferation activity of HUVECs in different groups. (D–E) Scratch wound healing assay results, scale bar = 100 μm. (F–G) Transwell migration assay results, scale bar = 100 μm. (H–I) Results of the tube formation assay, scale bar = 50 μm. (J) Relative expression of angiogenesis related mRNA in HUVECs (*Vegf, Pecam-1, Emcn*). (K–L) Relative expression of angiogenesis related proteins in HUVECs (VEGF, PECAM-1, EMCN), normalized to β-actin. ∗*P* < 0.05, ∗∗*P* < 0.01, ∗∗∗*P* < 0.001, each experiment was performed at least three times, one-way ANOVA with Tukey's multiple comparisons.

We also assessed the regulatory effect of the composite scaffold on angiogenesis using a multi-modal experimental approach (Fig. 3A). The results indicated that the GelMA/ICA@MSN/HAp composite scaffold extract significantly promoted the biological function of HUVECs. Specifically, the high-concentration ICA composite scaffold group exhibited a marked increase in cell proliferation (Fig. 3C). Live/Dead cell fluorescence imaging confirmed the formation of a dense endothelial monolayer on the scaffold surface (Fig. 3B). Furthermore, scratch wound healing and transwell migration assays revealed that the high-concentration ICA composite scaffold group exhibited significantly enhanced migratory capacity compared to other groups (Fig. 3D–G). The tube formation assay further demonstrated that the high-concentration ICA composite scaffold group promoted the development of a more complex tubular network, with a concentration-dependent increase in both branch node numbers and tube length (Fig. 3H and I). Mechanistic investigations revealed that the high-concentration ICA composite scaffold group significantly upregulated the proteins expression levels of key angiogenic differentiation markers, including vascular endothelial growth factor (VEGF), platelet endothelial cell adhesion molecule (PECAM-1), and endomucin-1 (EMCN). These results were corroborated by RT-qPCR analyses, which confirmed a dose-dependent increase in both protein and mRNA expression levels (Fig. 3J–L).

### 2.5 The GelMA/ICA@MSN/HAp composite scaffold promotes recruitment and lymphangiogenesis differentiation of LECs

**Fig. 4:**
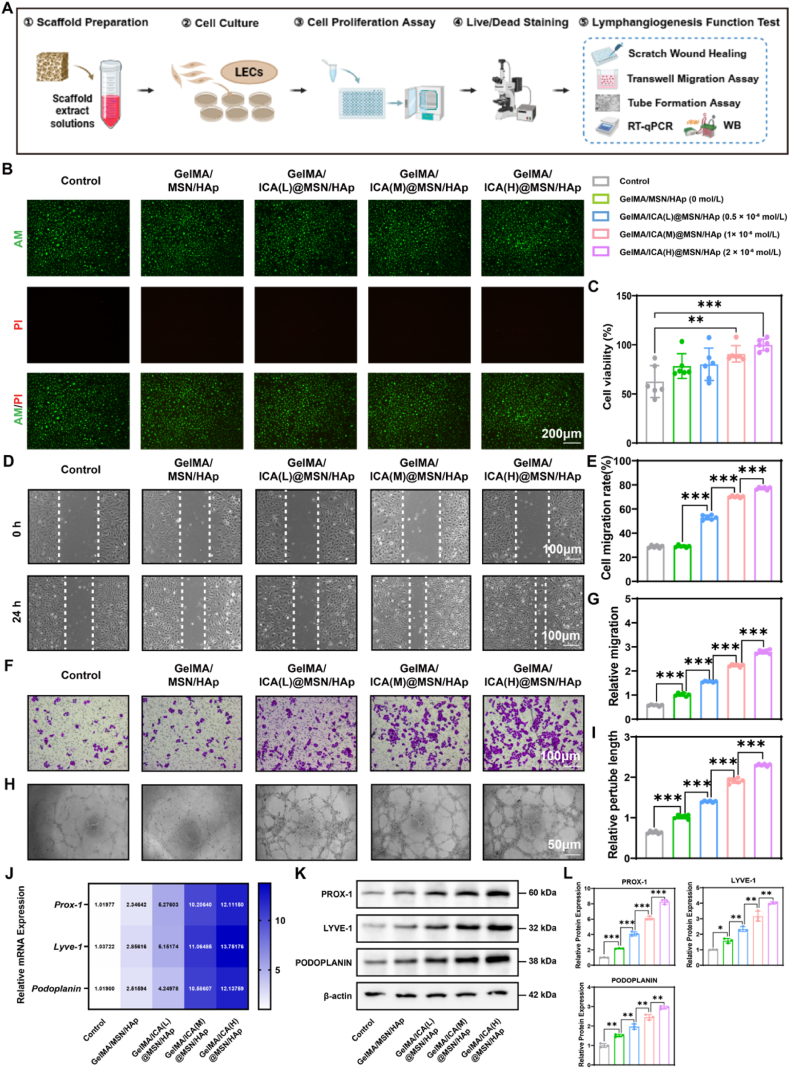
**GelMA/ICA@MSN/HAp composite scaffold promotes LECs proliferation and lymphangiogenesis differentiation.** (A) Schematic diagram illustrating the experimental design. (B) Inverted fluorescence microscope images of live/dead cells double fluorescent staining, scale bar = 200 μm. (C) Cell proliferation activity of LECs in different groups. (D–E) Results of the scratch wound healing assay, scale bar = 100 μm. (F–G) Results of the Transwell migration assay, scale bar = 100 μm. (H–I) Results of the tube formation assay, scale bar = 50 μm. (J) Relative expression of lymphangiogenesis related mRNA expression in LECs (*Prox-1, Lyve-1, Podoplanin*). (K–L) Relative expression of lymphangiogenesis related proteins in LECs (PROX-1, LYVE-1, PODOPLANIN), normalized to β-actin. ∗*P* < 0.05, ∗∗*P* < 0.01, ∗∗∗*P* < 0.001, each experiment was performed at least three times, one-way ANOVA with Tukey's multiple comparisons.

Lymphangiogenesis supports bone formation by enhancing the bone microenvironment and facilitating regenerative processes. To systematically evaluate the effect of the composite scaffold on lymphangiogenesis, a series of in vitro experiments were conducted (Fig. 4A). GelMA/ICA@MSN/HAp composite scaffold significantly enhanced the biological function of LECs. Notably, the high-concentration ICA scaffold group showed significantly increased cell proliferation compared to other groups (Fig. 4C). Live/Dead cell fluorescence imaging revealed the formation of a dense endothelial monolayer on the scaffold surface (Fig. 4B). Scratch wound healing and Transwell migration assays demonstrated that the high-concentration ICA scaffold group exhibited significantly greater cell migration efficiency than other groups (Fig. 4D–G). The tube formation assay confirmed that the high-concentration ICA composite scaffold facilitated the development of a more intricate lymphatic-like network structure, with a concentration-dependent increase in tube branching complexity (Fig. 4H and I). Molecular analysis further demonstrated that the high-concentration ICA composite scaffold group significantly up-regulated the protein expression levels of lymphangiogenic markers, including prospero homeobox 1 (PROX-1), lymphatic vessel endothelial receptor 1 (LYVE-1) and PODOPLANIN. These findings were validated by Western blot and RT-qPCR analyses, which confirmed a dose-dependent increase in both mRNA and protein expression levels (Fig. 4J–L).

### 2.6 Biocompatibility assessment of the GelMA/ICA@MSN/HAp composite scaffold

To evaluate the in vivo safety of the composite scaffold, a comprehensive biocompatibility assessment was performed using an osteoporotic femoral bone defect model. Experimental data indicated that serum biochemical markers across all groups (Model group, ICA group, GelMA/MSN/HAp group, and GelMA/ICA@MSN/HAp group) remained within the physiological reference range (Fig. S2A). Hematoxylin and eosin (H&E) staining further confirmed the absence of inflammatory cell infiltration or hepatic lobular structural disruption in liver tissues. Likewise, kidney histology revealed a well-defined boundary between the renal cortex and medulla, with intact glomeruli and renal tubule structures, showing no signs of pathological inflammation or structural abnormalities (Fig. S2B). Throughout the study period, the survival rate of the animals was 100 %, with no abnormal behavioral manifestations observed. Collectively, these findings demonstrate that the composite scaffold exhibits excellent systemic biocompatibility. Moreover, the process of its metabolic clearance exhibited no signs of toxicity to hepatic or renal parenchyma, indicating a favorable biosafety profile.

### 2.7 The GelMA/ICA@MSN/HAp composite scaffold promotes bone repair in vivo

**Fig. 5:**
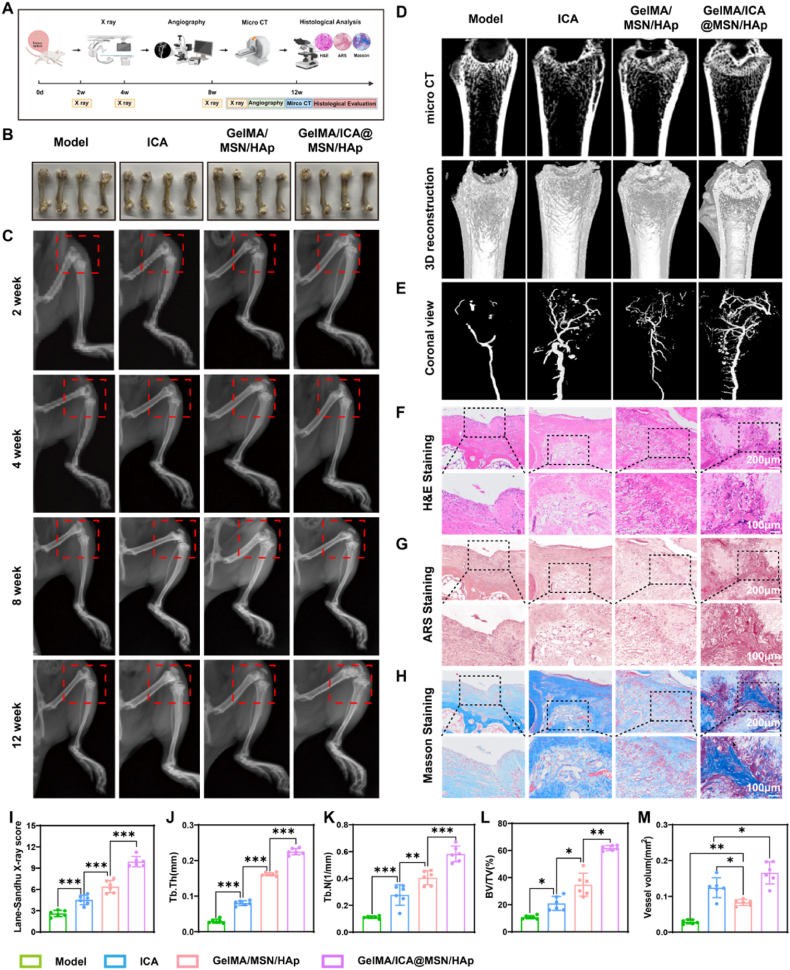
**GelMA/ICA@MSN/HAp composite scaffold facilitates bone repair in vivo model.** (A) Schematic diagram illustrating the experimental design. (B) Visual representation of bone defect repair. (C) X-ray examination of bone defect healing at different time points. (D) Micro CT analysis of bone repair. (E) Results of bone angiography. (F) H&E staining, scale bar = 200 μm or 100 μm. (G) Alizarin Red S staining, scale bar = 200 μm or 100 μm. (H) Masson staining, scale bar = 200 μm or 100 μm. (I) Statistical analysis of Lane-Sandhu scores. (J) Quantitative analysis of Tb.Th quantitative parameters. (K) Quantitative analysis of Tb.N quantitative parameters. (L) Quantitative analysis of BV/TV quantitative parameters. (M) Quantitative analysis of angiography. ∗*P* < 0.05, ∗∗*P* < 0.01, ∗∗∗*P* < 0.001, each experiment was performed at least three times, one-way ANOVA with Tukey's multiple comparisons.

SD rats were implanted with scaffold at the femur bone defect site for 12 weeks, and we assess the situation of bone repair at different time point (Fig. 5A). Dynamic monitoring over 12 weeks post-surgery confirmed that no local tissue necrosis or systemic toxicity was observed in any experimental group (Fig. 5B), demonstrating the material's excellent biocompatibility and tissue compatibility. The experimental results revealed a hierarchical pattern of bone regeneration efficacy, with the GelMA/ICA@MSN/HAp composite scaffold group exhibiting significantly superior bone regeneration quality, speed, and maturity compared to other groups (Model group < ICA group < GelMA/MSN/HAp group < GelMA/ICA@MSN/HAp group) (Fig. 5C and D). Furthermore, Lane-Sandhu scores for the GelMA/ICA@MSN/HAp scaffold group were significantly higher than other group, which, in turn, outperformed the ICA group (Fig. 5I). Micro-CT quantitative analysis of bone parameters further demonstrated composite scaffold have higher Tb.Th, Tb.N and BV/TV(Fig. 5J–L).

Histopathological analysis provided further insight into the scaffold's osteogenic potential. H&E staining revealed that the GelMA/ICA@MSN/HAp composite scaffold group formed continuous lamellar bone structures at the defect site, with intact bone lacunae and canaliculi. In contrast, the Model group primarily exhibited fibrous bone callus, while the ICA group displayed disorganized woven bone formation (Fig. 5F). ARS staining confirmed that the composite scaffold group exhibited mature concentric mineralization patterns, whereas the ICA group retained punctate immature bone-like tissue (Fig. 5G). Additionally, Masson staining demonstrated significantly better collagen fiber alignment and cortical bone reconstruction integrity in the composite scaffold group, suggesting a bone regeneration microenvironment that more closely mimics physiological bone remodeling (Fig. 5H).

Angiographic quantification demonstrated significant differences in neovascular density among the experimental groups (Model group < GelMA/MSN/HAp group < ICA group < GelMA/ICA@MSN/HAp group) (Fig. 5E–M). Compared to the Model group, the GelMA/MSN/HAp composite scaffold promoted baseline angiogenesis, while the ICA group, through its bioactive components, significantly enhanced vascular maturity. The GelMA/ICA@MSN/HAp composite scaffold group achieved optimal angiogenic branching complexity and endothelial connectivity by integrating material-mediated physical guidance with ICA-controlled drug release, resulting in a denser and functionally stable neovascular network.

### 2.8 The GelMA/ICA@MSN/HAp composite scaffold facilitates tripartite coordination of osteogenesis, angiogenesis and lymphangiogenesis in a bone defect model

**Fig. 6:**
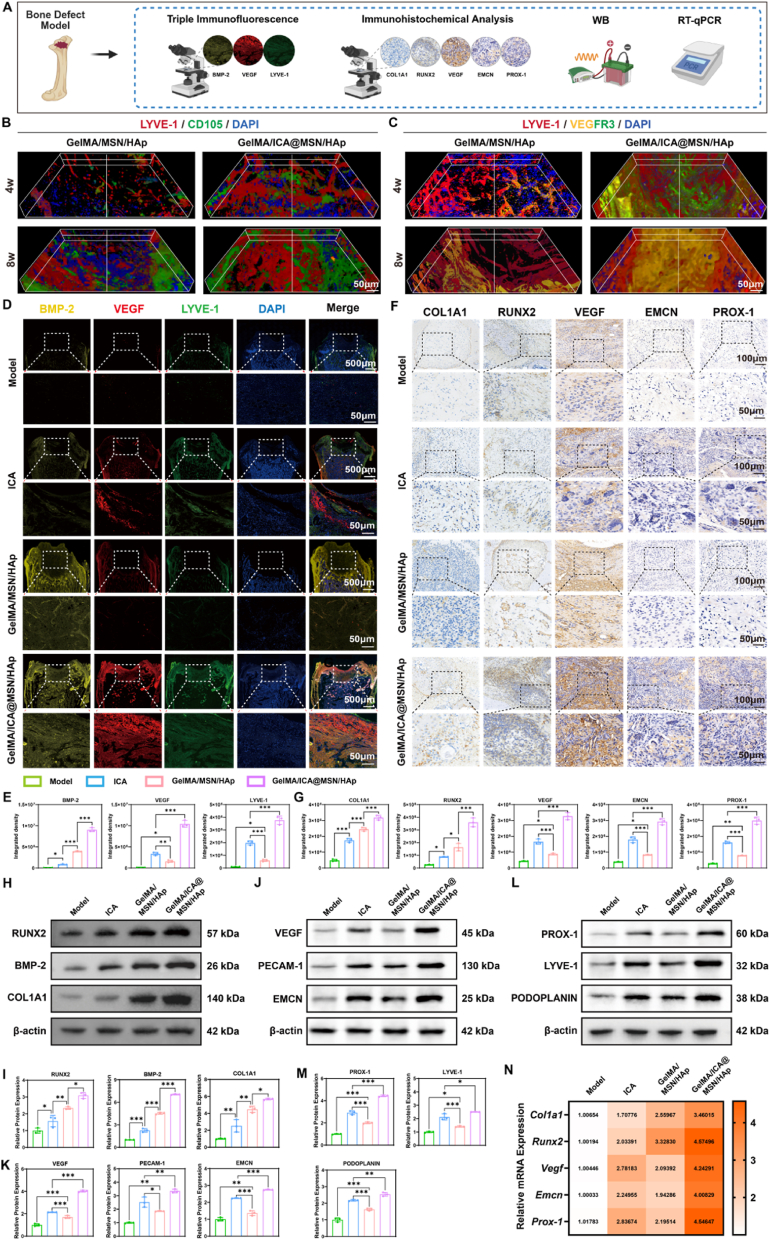
**GelMA/ICA@MSN/HAp composite scaffold facilitates tripartite coordination of osteogenesis, angiogenesis and lymphangiogenesis.** (A) Schematic diagram illustrating the experimental design. (B) 3D reconstruction showing the expression of LYVE-1 (an LECs marker) and CD105 (a BMSCs marker) in the bone repair site at 4 and 8 weeks post-implantation of the composite scaffold. Scale bar = 50 μm. (C) 3D reconstruction showing the expression of LYVE-1 (an LECs marker) and VEGFR3 (an HUVECs marker) in the bone repair site at 4 and 8 weeks post-implantation of the composite scaffold. Scale bar = 50 μm. (D, E) Triple immunofluorescence staining demonstrating the co-localization of BMP-2, VEGF, and Lyve-1 at the bone repair site, scale bar = 500 μm or 50 μm. (F, G) Immunohistochemical analysis illustrating the progressive regulatory characteristics of osteogenic-vascular-lymphangiogenic regeneration, scale bar = 100 μm or 50 μm. (H, I) Protein expression of osteogenesis related factors (RUNX2, BMP-2, COL1A1). (J, K) Protein expression of angiogenesis related factors (VEGF, PECAM-1, EMCN). (L, M) Protein expression of lymphangiogenesis related factors (PROX-1, LYVE-1, PODOPLANIN). (N) mRNA expression of osteogenesis-related factors (*Col1a1, Runx2, Vegf, Emcn, Prox-1*). ∗*P* < 0.05, ∗∗*P* < 0.01, ∗∗∗*P* < 0.001, each experiment was performed at least three times, one-way ANOVA with Tukey's multiple comparisons.

We then systematically investigated the coordination regulatory mechanisms by which the GelMA/ICA@MSN/HAp composite scaffold remodels the bone microenvironment (Fig. 6A).

3D reconstruction revealed that the GelMA/ICA@MSN/HAp group exhibited significantly higher expression of LYVE-1, VEGFR3, and CD105 than the GelMA/MSN/HAp group at both 4 and 8 weeks. A temporal analysis further showed that the expression of these markers increased markedly from week 4 to week 8 within the GelMA/ICA@MSN/HAp group, indicating a sustained enhancement in the recruitment of LECs, HUVECs, and BMSCs (Fig. 6B and C). Triple immunofluorescence analysis revealed a significant increase in VEGF and LYVE-1 co-localization across all treatment groups compared to the model group, with the GelMA/ICA@MSN/HAp composite scaffold exhibiting the most pronounced lymphangiogenic activity. Additionally, BMP-2 fluorescence intensity peaked in the composite scaffold group, indicating that the material's sustained-release properties maintained the prolonged bioactivity of this key osteogenic factor (Fig. 6D and E).

Immunohistochemical analysis further delineated the hierarchical differences in osteogenic differentiation among the groups. Compared to the ICA group, the GelMA/MSN/HAp composite scaffold significantly up-regulated COL1A1 and RUNX2 expression. Notably, the GelMA/ICA@MSN/HAp composite scaffold exhibited an even larger COL1A1-positive area and higher RUNX2 nuclear translocation efficiency, indicating that ICA synergistically enhanced the scaffold's molecular regulatory network. Furthermore, the ICA group exhibited significantly higher expression of Endomucin and VEGF than GelMA/MSN/HAp group, highlighting ICA's capacity to promote vascularization. The GelMA/ICA@MSN/HAp composite scaffold further amplified the expression of these markers through the combined effects of material-controlled drug release and ICA bioactivity, leading to improved vascular network stability. In terms of lymphangiogenesis, Prox-1 expression was significantly higher in the ICA group than in the GelMA/MSN/HAp group. Notably, the GelMA/ICA@MSN/HAp composite scaffold, through mechanical microenvironment guidance and spatiotemporal drug release synergy, induced the co-expression of PROX-1, LYVE-1, and PODOPLANIN, thereby significantly enhancing the structural maturity of lymphatic vessel lumens (Fig. 6F and G).

Furthermore, the protein and mRNA expression demonstrated a progressive upregulation of osteogenic markers, including RUNX2, BMP-2 and COL1A1, with the GelMA/ICA@MSN/HAp composite scaffold group exhibiting the highest expression levels, suggesting that its controlled-release system and bioactive components synergistically enhanced osteogenesis (Fig. 6H, I, N). Similarly, angiogenesis-related markers (VEGF, PECAM-1 and EMCN) followed a hierarchical trend, with the composite scaffold group displaying the strongest vascularization potential (Fig. 6J, K, N). Lymphangiogenesis-specific markers (PROX-1, LYVE-1 and PODOPLANIN) also reached peak expression in the composite scaffold group, further confirming its superior ability to promote lymphatic vessel formation (Fig. 6L, M, N).

### 2.9 GelMA/ICA@MSN/HAp composite scaffold activate mitochondrial metabolic reprogramming during the bone repair process

**Fig. 7:**
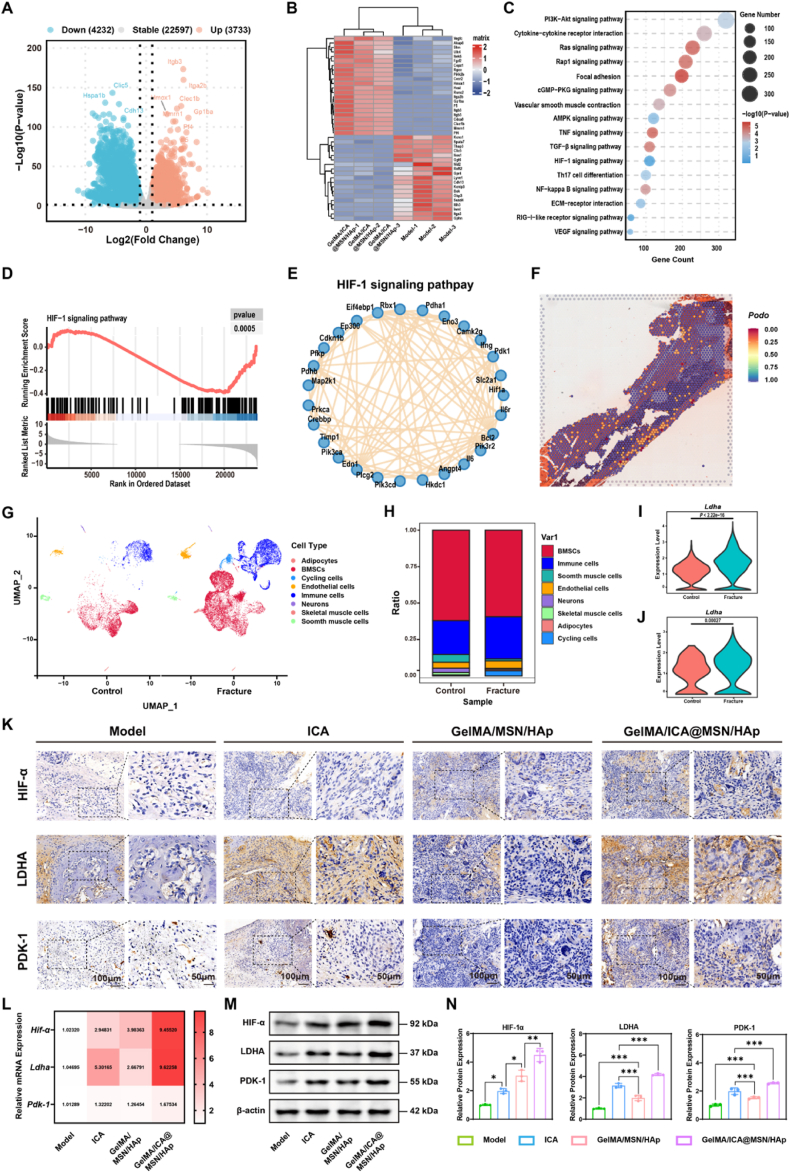
**GelMA/ICA@MSN/HAp composite scaffold promotes osteogenesis through lymphatic activation and HIF-1α mediated mitochondrial metabolic reprogramming.** (A) Volcano plot highlighting differentially expressed genes between groups. (B) Heatmap clustering analysis of highly expressed characteristic genes. (C) Functional enrichment analysis of biological processes. (D) GSEA confirming the enrichment of key signaling pathways. (E) HIF-1 signaling pathway core node gene expression level verification. (F) stRNA-seq analysis showing the distribution and expression of the lymphatic marker PDPN around fracture sites. (G, H) scRNA-seq analysis cell populations in control and fracture models. (I, J) scRNA-seq analysis of Ldha mRNA expression in BMSCs (I) and HUVECs (J). (K) Immunohistochemical (IHC) staining for HIF-1α, LDHA and PDK-1 in bone tissue, scale bar = 100 μm or 50 μm. (L) Relative mRNA expression levels of Hif-1α, Ldha and Pdk-1 in bone tissue. (M, N) Protein expression levels of HIF-1α, LDHA and PDK-1 in bone tissue. ∗*P* < 0.05, ∗∗*P* < 0.01, ∗∗∗*P* < 0.001, each experiment was performed at least three times, one-way ANOVA with Tukey's multiple comparisons.

To further investigate the molecular mechanisms underlying the bone repair effects of the GelMA/ICA@MSN/HAp composite scaffold, transcriptome sequencing was performed. The Results showed that 81.8 % of genes were shared between the GelMA/ICA@MSN/HAp and Model groups, while 9.5 % of genes were uniquely expressed in the composite scaffold group. In total, 7965 significantly altered genes were identified, with notable upregulation of pro-angiogenic markers such as Itgb3 and Itga2b, and downregulation of Clic5 and Hspa1b (Fig. 7A). Heatmap clustering further confirmed the higher expression of genes related to angiogenesis and cell migration in the composite scaffold group (Fig. 7B).

GO and GSEA analysis revealed significant involvement of the HIF-1 and VEGF signaling pathways (Fig. 7C and D), with key regulatory genes such as *Hif-1ɑ* highly expressed in the composite scaffold group (Fig. 7E). Additionally, we leveraged public scRNA-seq and spatial-temporal transcriptome sequencing (stRNA-seq) datasets from normal and osteoporotic fracture models. stRNA-seq analysis demonstrated that the lymphatic vessel marker Podoplanin showed increased distribution and expression around the fracture repair sites (Fig. 7F).

scRNA-seq analysis further revealed an accumulation of immune cells and endothelial cells at the osteoporotic fracture sites (Fig. 7G and H), accompanied by upregulation of mitochondrial energy metabolism-related genes such as *Ldha* (Fig. 7I and J). Collectively, these findings indicate that lymphatic endothelial cells play a crucial role in the bone repair process. Moreover, IHC analysis revealed higher expression levels of HIF-1α, LDHA and PDK-1 in the composite scaffold group compared to the GelMA/MSN/HAp group (Fig. 7K), consistent with the trends observed at both the protein and mRNA levels (Fig. 7L–N). Taken together, these results suggest that mitochondrial metabolic reprogramming is activated during the bone repair process mediated by the composite scaffold.

### 2.10 LECs regulate glycolysis and mitochondrial metabolic reprogramming via HIF-1α signaling

**Fig. 8:**
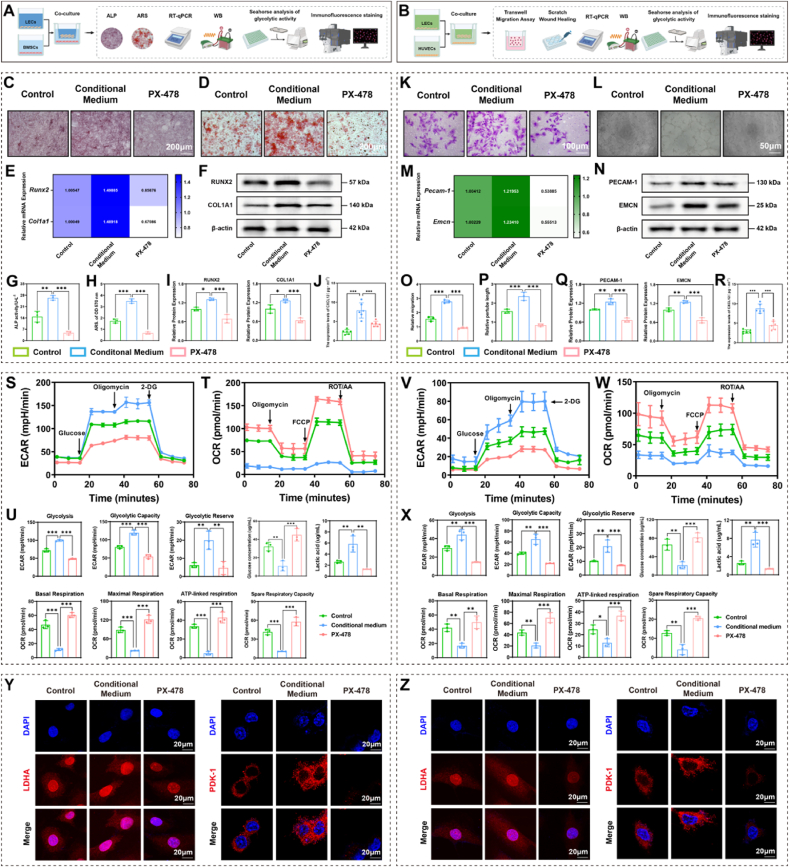
**LECs regulate osteogenesis, angiogenesis, and metabolic reprogramming via HIF-1α signaling.** (A, B) Schematic diagram illustrating the experimental design. (C, G) ALP staining and quantitative analysis, scale bar = 200 μm. (D, H) ARS staining and quantitative analysis, scale bar = 200 μm. (E) mRNA expression of osteogenesis-related factors (*Runx2, Col1a1*). (F, I) Protein expression of osteogenesis related factors (RUNX2, COL1A1). (J, R) CXCL12 expression in different groups. (K, O) Transwell migration assay results, scale bar = 100 μm. (L, P) Results of the tube formation assay, scale bar = 50 μm. (M) mRNA expression of vascularization-related factors (*Pecam-1, Emcn*). (N, Q) Protein expression of vascularization-related factors (PECAM-1, EMCN). (S) ECAR in BMSCs. (T) OCR in BMSCs. (U) Glycolytic and mitochondrial parameters in BMSCs. (V) ECAR in HUVECs. (W) OCR in HUVECs. (X) Glycolytic and mitochondrial parameters in HUVECs. (Y) Immunofluorescence staining of glycolytic markers in BMSCs (LDHA, PDK-1). (Z) Immunofluorescence staining of glycolytic markers in HUVECs (LDHA, PDK-1). ∗*P* < 0.05, ∗∗*P* < 0.01, ∗∗∗*P* < 0.001, each experiment was performed at least three times, one-way ANOVA with Tukey's multiple comparisons.

Previous findings suggested that LECs play a critical role in bone repair and may influence the behavior of BMSCs and HUVECs. To further explore these interactions, we conducted co-culture experiments in which LECs were incubated with either BMSCs or HUVECs (Fig. 8A and B). The results showed that conditional medium derived from LECs significantly enhanced the osteogenic potential of BMSCs, as evidenced by increased calcium nodule formation, elevated ALP activity, and upregulated expression of osteogenic markers RUNX2 and COL1A1. However, these effects were markedly attenuated following treatment with PX-478, a specific HIF-1α inhibitor (Fig. 8C–I). Similarly, in HUVECs, conditional medium significantly promoted migratory ability, increased tube density, and enhanced tubular complexity, accompanied by elevated expression of endothelial markers ECAM-1 and EMCN (Fig. 8K–Q). The ELISA results showed that CXCL12 expression was lowest in the control group under baseline conditions. It was significantly increased in the conditional medium group, demonstrating the composite scaffold's promotive effect, and markedly reduced by the PX-478 inhibitor, respectively. Both changes were statistically significant (Fig. 8J–R). Together, these results demonstrate that LECs-conditional medium, particularly after treatment with the composite scaffold, promotes both osteogenesis of BMSCs and angiogenesis of HUVECs through mechanisms involving HIF-1α signaling.

Given the observed mitochondrial metabolic changes during bone repair, we next assessed glycolytic activity in BMSCs and HUVECs. The extracellular acidification rate (ECAR), an indicator of glycolytic flux, was significantly increased in the conditioned medium group compared to controls, suggesting enhanced glycolysis. Treatment with PX-478 reversed this increase, implicating HIF-1α as a key regulator (Fig. 8S–U, V, X). Consistently, oxygen consumption rate (OCR) assays showed suppressed mitochondrial respiration following conditioned medium treatment, with partial recovery upon PX-478 administration (Fig. 8T, U, W, X).

Immunofluorescence staining further revealed that conditioned medium elevated the expression and distribution of glycolysis-related markers LDHA and PDK-1 in both BMSCs and HUVECs (Fig. 8Y and Z). Collectively, these results indicate that LECs regulate glycolysis and mitochondrial metabolic reprogramming via HIF-1α signaling, thereby contributing to enhanced bone repair.

## 3 Discussion

We developed a novel 3D-printed GelMA/ICA@MSN/HAp scaffold that orchestrates osteogenic, angiogenic, and lymphogenic coupling, leading to superior and spatially organized bone regeneration in vivo. This study thereby establishes a new paradigm for bone regeneration by demonstrating the efficacy of this triple-coupling strategy. Our work makes two key advances: it introduces the lymphatic system as an active regulator in bone repair, moving beyond its traditional passive role, and identifies HIF-1α mediated mitochondrial metabolic reprogramming as the core mechanism through which scaffold-activated lymphatic endothelial cells coordinately enhance osteogenesis and angiogenesis. These findings redefine the landscape of multifunctional biomaterials by incorporating lymphatic instruction as a central design principle.

Bone regeneration is a highly coordinated process involving interactions between bone tissue, vascularization, and lymphangiogenesis [35]. Our scRNA-seq analysis further confirmed significant changes in the BMSCs and endothelial cell clusters between control and fracture bone tissue. In vitro, the GelMA/ICA@MSN/HAp composite scaffold significantly enhanced the osteogenic differentiation of BMSCs, as evidenced by increased ALP activity, intensified ARS staining and upregulation of osteogenic markers such as RUNX2, COL1A1, and BMP-2 at both the mRNA and protein levels. Simultaneously, the scaffold facilitated the adhesion and migration of HUVECs and LECs, promoting the formation of a functional vascular-lymphatic network, with significantly elevated expression of VEGF, PECAM-1, EMCN, PROX-1, and LYVE-1. In vivo, the scaffold exhibited excellent biocompatibility without apparent hepatotoxicity or nephrotoxicity. It effectively promoting new bone formation, angiogenesis, and lymphangiogenesis at the defect site. Immunohistochemical staining revealed the strongest positive signals for RUNX2, COL1A1, and BMP-2 in the GelMA/ICA@MSN/HAp group, corroborated by Western blot and RT-qPCR analyses, indicating a more pronounced osteogenic effect compared to ICA gavage alone or GelMA/MSN/HAp implantation. It is worth noting that the GelMA/MSN/HAp scaffold also promoted the proliferation of HUVECs and LECs, as well as angiogenesis and lymphangiogenesis. This effect may be attributed to the release of calcium and phosphate ions, which have been previously shown to enhance vascular and lymphatic growth [36,37]. Taken together, the GelMA matrix not only provided a biomimetic 3D structure but also preserved RGD sequences that supported the adhesion, proliferation, and migration of BMSCs, HUVECs, and LECs. These recruited cell further self-organized into “cell islands,” creating a dynamic microenvironment that actively promoted bone regeneration. This self-assembly behavior underscores the scaffold's ability to coordinate osteogenesis, angiogenesis, and lymphangiogenesis, offering a promising strategy for advanced bone tissue engineering.

To further elucidate the underlying mechanisms, transcriptomic sequencing and subsequent bioinformatic analyses confirmed the multifaceted regulatory effects of the GelMA/ICA@MSN/HAp composite scaffold. Differential gene expression patterns were observed across multiple signaling pathways involved in bone repair, particularly HIF-1 and VEGF. Generally, HIF-1 signaling is known to enhance osteogenic cell survival under hypoxic conditions, promoting bone matrix synthesis and deposition [38,39]. Concurrently, upregulation of VEGF expression promoted neovascularization, improving the supply of oxygen and nutrients essential for tissue regeneration [40,41]. More importantly, recent studies have highlighted that HIF-1 signaling plays a pivotal role in mitochondrial energy metabolism. In our study, the composite scaffold group showed higher distribution, protein, and mRNA levels of glycolysis-related markers LDHA and PDK-1 compared to the GelMA/MSN/HAp group. Additionally, scRNA-seq data indicated increased *Ldha* expression in fractured bone tissue relative to controls. These findings suggest that bone tissue undergoes mitochondrial metabolic reprogramming during the repair process, aligning with previous studies indicating that enhanced glycolysis promotes bone fracture healing [42].

Recent studies have emphasized the importance of the microenvironment in bone repair, particularly the role of lymphatic regulation in osteogenic-angiogenic coupling during bone regeneration [43]. Conditional medium from LECs pre-treated with the composite scaffold significantly stimulated osteogenesis of BMSCs and angiogenesis of HUVECs. These effects were inhibited by PX-478, a specific HIF-1α inhibitor, confirming the centrality of this pathway. Moreover, both OCR (reflecting mitochondrial respiration) and ECAR (indicating glycolytic activity) were markedly affected by LECs [44], supporting the role of metabolic reprogramming in these processes. These observation align with previous reports indicating reduced in osteoporotic patients and that enhanced glycolysis in BMSCs facilitates osteogenic differentiation [45]. Additionally, accelerating aerobic glycolysis has been shown to promote angiogenesis in HUVECs [46]. Collectively, these findings demonstrate that the GelMA/ICA@MSN/HAp scaffold establishes a favorable microenvironment for osteogenesis and synchronized vascular-lymphatic regeneration, thereby significantly enhancing the efficiency and quality of bone repair. This strategy offers a promising avenue for addressing osteoporotic bone defects and advancing bone tissue engineering. At the same time, this study has certain limitations. Although the lymphatic system is essential in bone repair, we focused primarily on LECs and did not explore the potential contributions of broader lymphatic transport or vascular-lymphatic crosstalk. In addition, the therapeutic efficacy of the scaffold was not evaluated in a larger animal model, which will be necessary for future clinical translation.

## 4 Methods

See supplementary files for details.

## CRediT authorship contribution statement

**Weipeng Sun:** Writing – original draft, Methodology, Conceptualization. **Qing Lin:** Methodology, Investigation. **Biyi Zhao:** Writing – original draft, Methodology. **Minying Li:** Writing – original draft, Formal analysis. **Jiacong Xiao:** Resources, Methodology. **Xueshan Jin:** Supervision. **Jinfu Liu:** Validation. **Yifei Wang:** Investigation, Formal analysis. **Ronghua Zhang:** Writing – review & editing, Funding acquisition. **Xiaoyun Li:** Writing – review & editing, Project administration, Funding acquisition. **Ziwei Jiang:** Writing – review & editing, Validation, Funding acquisition.

## Ethics approval and consent to participate

The Animal Experiment Protocol listed has been reviewed and approved by Laboratory Animal Welfare and Ethics Committee of Jinan University (approval number: IACUC-20250107-04).

## Declaration of competing interest

The authors declare no conflict of interest.

## Figures and Tables

**figs1:**
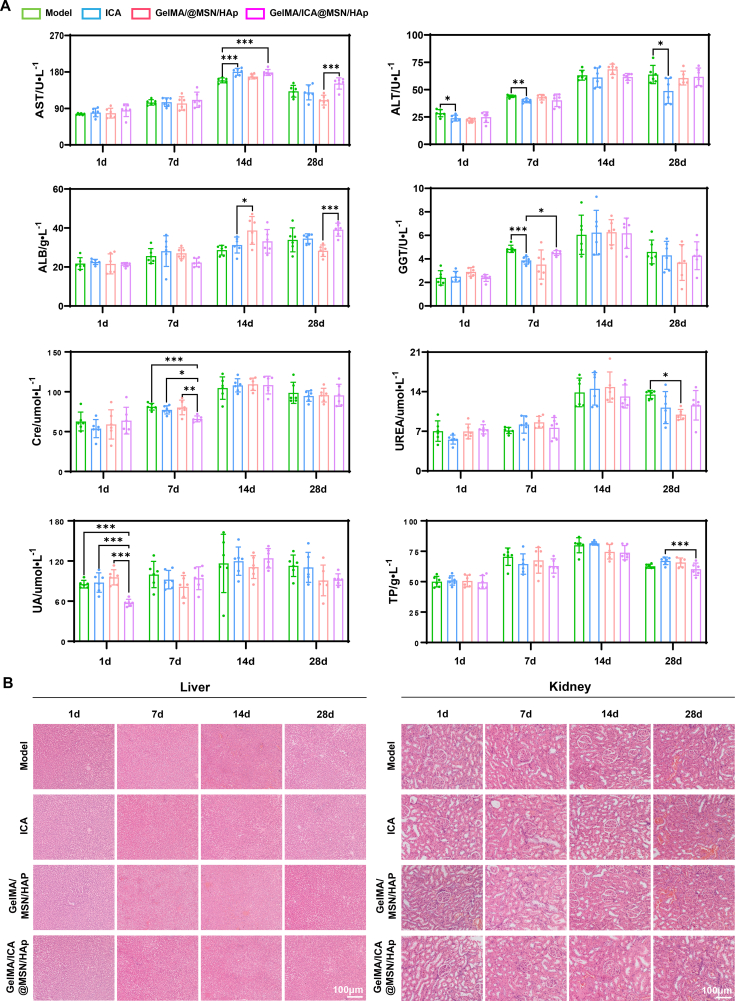


**figs2:**