# Supplementary material for: Lymphatic incorporated biomimetic scaffold enhances Osteoangio-lymphogenic coupling via HIF-1α mediated mitochondrial reprogramming for osteoporotic bone repair
Source: Bioact Mater. 2025 Nov 4;56:641–55. doi: 10.1016/j.bioactmat.2025.10.041 (PMC12636390; doi:10.1016/j.bioactmat.2025.10.041)
Supplement: Multimedia component 1 [file mmc1.docx]

**Materials and Methods**

***1. Material Synthesis and Characterization Procedures***

***1.1Preparation of Icariin-Loaded Mesoporous Silica***

To prepare icariin (ICA)-loaded mesoporous silica (ICA@MSN), 100 mg of MSN powder was dispersed in 10 mL of anhydrous ethanol and sonicated to achieve uniform dispersion. Subsequently, 100 mg of ICA was added to the suspension and stirred until fully dissolved. The mixture was then stirred overnight on ice to facilitate drug loading. Following incubation, the suspension was centrifuged at 8,000 rpm for 10 min to collect the precipitate. The supernatant was washed 2-3 times with phosphate-buffered saline to remove unbound ICA. Finally, the ICA@MSN was freeze-dried for further use.

***1.2 Preparation composite scaffold***

GelMA was dissolved in deionized water containing a specific ratio of lithium phenyl-2,4,6-trimethylbenzoylphosphinate (LAP) as a photoinitiator and Fast Green FCF as a photo-absorber to facilitate the printing of small pore sizes. The photoinitiator solution was prepared by dissolving 1 mg of LAP and 0.6 mg of Fast Green FCF in 1 mL of deionized water, followed by vortex mixing for 30 s. Subsequently, 0.15 g of GelMA was dissolved in the prepared solution to obtain a 15% GelMA solution. To enhance the bioactivity of the hydrogel, 10 mg of hydroxyapatite (HAp) and 3 mg of ICA@MSN composite were added to the mixture. The prepared bio-ink was loaded into a 3D printer, and printing was performed under the following conditions: light intensity of 17 mW/cm², exposure time of 20 s, base layer number of 1, and base layer exposure time of 27 s. After printing, the hydrogel constructs were rinsed 2-3 times with sterile phosphate-buffered saline to obtain the final hydrogel scaffold.

***1.3 Characterization of scaffold***

The particle size distribution and zeta potential of the nanoparticles were measured using a laser nanoparticle size analyzer. Their morphology was examined via transmission electron microscopy (TEM). Structural and compositional analyses were conducted using X-ray diffraction (XRD) and Fourier-transform infrared spectroscopy (FTIR). Magnetic properties were assessed using a vibrating sample magnetometer (VSM). The microstructure of the hydrogels was observed by scanning electron microscopy (SEM), and elemental distribution was analyzed using energy-dispersive X-ray spectroscopy (EDS). Icariin loading capacity and encapsulation efficiency were quantified using a UV-Vis spectrophotometer. The rheological properties of the scaffold were evaluated using a rheometer. In addition, the swelling behavior and degradation profile of the hydrogels were assessed in subsequent experiments.

***2. Cell Experiments***

#### *2.1 Preparation Of Scaffold Extracts*

#### Based on the effective concentration of icariin (ICA) (1×10^-6^ mol/L) used in previous studies, this was used as the middle concentration for setting low and high concentrations. ICA was dissolved in DMSO and diluted to 0.5×10^-6^ mol/L, 1×10^-6^ mol/L, and 2×10^-6^ mol/L. This was used to prepare 3D printing slurries, and subsequently, 10 mm × 10 mm composite drug-loaded scaffolds and blank scaffolds without drug were prepared. Based on the ICA loading concentration, the scaffolds were divided into four groups: blank scaffold group, low-concentration drug-loaded scaffold group [GelMA/ICA(L)@MSN/HAp] (0.5×10^-6^ mol/L), middle concentration drug-loaded scaffold group [GelMA/ICA(L)@MSN/HAp] (1×10^-6^ mol/L), and high concentration drug-loaded scaffold group [GelMA/ICA(L)@MSN/HAp] (2×10^-6^ mol/L). Subsequently, each group of scaffolds was extracted at an extraction ratio of 1 mg/mL for 24 h, and the extract was collected for subsequent experiments.

#### *2.1 Cell Proliferation Assay*

#### Cell proliferation assay was conducted to evaluate the viability of HUVECs, BMSCs and LECs and to assess the cytotoxicity of scaffold extracts. Cells were seeded into 96-well plates at a density of 5,000 cells per well. After incubation for 24, 48 and 72 h, 10 μL of cell counting kits 8 (CCK-8) solution (Ab228554, Abcam) was added to each well. The plates were then incubated for 2 h at 37 °C in dark. Absorbance was measured at 460 nm using a microplate reader.

#### *2.2 Alkaline Phosphatase (ALP) Assay*

BMSCs were seeded at 5 × 10⁴ cells per well in 24-well plates, cultured for 24 h in α-MEM medium. After 24 h, the medium was replaced with scaffold extracts, and continue cultured for 14 d, with media changes every 72 h. The cells were then fixed with 4% paraformaldehyde for 30 min at room temperature. ALP staining (C3206, Beyotime, China) was performed by incubating the fixed cells with the staining solution for 30 min in dark at room temperature. Following staining, cells were washed three times with PBS, each wash lasting 5 min.

#### *2.3 Alizarin Red S (ARS) Staining*

BMSCs were seeded at a density of 5 × 10⁴ cells per well in 24-well plates and cultured for 24 h in α-MEM medium. The medium was then replaced with scaffold extract solutions, and continue cultured for 21 d, with regular media changes. After incubation, cells were fixed with 4% PFA and washed three times with distilled water. The cells were then stained with Alizarin Red S solution for 30 min at 37°C. Stained mineralized nodules were observed and imaged under a microscope.

#### *2.4 Transwell Migration Assay*

HUVECs and LECs were seeded at a density of 2 × 10^4^ cells in the upper chamber of 24-well Transwell plates with an 8 μm pore size. The lower chamber contained 500 μL of extract solution. After 24 h of incubation, non-migrated cells on the upper membrane surface were removed, and the migrated cells were fixed with 4% PFA for 30 min. After staining with 1% crystal violet for 10 min, cells were observed under an inverted microscope (IX51, Olympus). The experiment was repeated three times for reproducibility.

#### *2.5 Tube Formation Assay*

HUVECs and LECs were cultured at 2 × 10^4^ cells per well onto Matrigel-coated 96-well plates (50 μL) for 12 h in scaffold extract solutions. After incubation, morphological changes in the cells were observed under a microscope. The length of the formed tubes was measured using ImageJ software.

#### *2.6 Live/Dead Staining analysis*

### BMSCs, HUVECs and LECs were seeded in appropriate culture plates or slides and cultured under experimental conditions. After the incubation period, cells were washed with PBS to remove remaining medium and suspended cells. Live/dead staining was performed using Calcein AM (green fluorescence) to stain live cells and propidium iodide (PI, red fluorescence) to stain dead cells. After incubation at room temperature for 30 min, the cells were washed with PBS and observed under a fluorescence microscope. The fluorescence intensities of live and dead cells were analyzed for cell viability assessment.

#### *2.7 Western Blot*

Sample were homogenized in lysis buffer containing protease and phosphatase inhibitors. The homogenates were centrifuged at 12,000 rpm for 10 min at 4 °C, and the supernatant was collected. Protein concentration was determined using a BCA protein assay kit. Proteins were separated by Sodium dodecyl-sulfate polyacrylamide gel electrophoresis and transferred onto polyvinylidene fluoride membranes. The membranes were then blocked with 5% skim milk for 1 h at room temperature, followed by overnight incubation at 4 °C with primary antibodies against HIF-1α (bsm-62518R, 1: 1000, Bioss), RUNX2 (GB115631-100, 1: 1000, Seville), BMP-2 (bs-1012R, 1: 1000, Bioss), COL1A1 (GB115707-100, 1: 1000, Seville), β-actin (bs-0061R, 1: 5000, Bioss), VEGF (bs-0170R, 1: 1000, Bioss), PECAM-1 (bsm-52428R, 1: 1000, Bioss), EMCN (bs-5884R, 1: 1000, Bioss), PROX-1 (bs-2774R, 1: 1000, Bioss), LYVE-1 (bs-1311R, 1: 1000, Bioss) and PODOPLANIN (bsm-52936R, 1: 1000, Bioss), LDHA (bsm-62875R, 1:1000, Bioss)、PDK-1(bs-3788R, 1:1000, Bioss). After washing, the membranes were incubated with HRP-conjugated secondary antibodies, and protein bands were visualized using enhanced chemiluminescence reagents. Quantification was performed based on the gray intensity values of the bands.

#### *2.8 Real-Time quantitative polymerase chain reaction (RT-qPCR) analysis*

Total RNA was extracted using the Takara Total RNA Extraction Kit, and its concentration and purity were assessed with a NanoDrop 2000 spectrophotometer. Reverse transcription was performed with the PrimeScript™ RT Kit (Takara), followed by qPCR using Premix Ex Taq™II. The reaction conditions included an initial denaturation at 95 °C for 30 s, followed by 40 cycles of 5 s at 95 °C and 30 s at 60 °C. β-actin was used as the housekeeping gene for normalization, and relative mRNA expression levels were analyzed using the 2^−ΔΔCT^ method. Primer sequences used for qPCR are listed in **Table 1**.

***3. Animal Experiments***

#### *3.1 Animal treatment*

#### Animal experiments strictly follow the 3R principle. A total of 64 adult Sprague-Dawley (SD) rats, weighing between 280-300 g, from Zhuhai Bestest Bio-Tech Co., Ltd. (SCXK (Yue) 2020-0051), were used in this study. To ensure randomization, the rats were divided into four groups using a random number table: the femoral bone defect model group (model group), ICA gavage group (ICA group), femoral condyle bone defect implanted with a blank GelMA/MSN/HAp scaffold group (GelMA/MSN/HAp group), and femoral condyle bone defect implanted with an ICA-loaded GelMA/MSN/HAp scaffold group (GelMA/ICA@MSN/HAp group). The animal study followed the National Research Council guide for the care and use of laboratory animals, and was approved by the Animal Care Committee of Jinan University [Experimental Ethics No. IACUC-20250107-04].

#### *3.2 Model Construction and Postoperative Care*

To simulate postmenopausal osteoporosis, osteoporotic rat models were established via bilateral ovariectomy. Ovariectomy procedure: Rats were anesthetized with 3% sodium pentobarbital via intraperitoneal injection, then fixed and disinfected. A 0.5–1.0 cm incision was made approximately 1 cm lateral to the posterior midline of the dorsal rib arch to expose the ovaries. The ovaries were ligated and excised, followed by suturing of the incision. Postoperatively, intramuscular penicillin was administered to prevent infection. Rats were housed under standard conditions for 12 weeks, after which bone mineral density (BMD) was measured to confirm successful osteoporosis induction. Successful model rats were weighed and anesthetized with 3% sodium pentobarbital at a dose of 45 mg/kg. The rats were fixed in a supine position and their skin over the distal femur was disinfected with iodine. 2 mm drill bit was used to create 2 mm diameter, 2 mm deep cylindrical defect on the bone surface, followed by saline lavage to cool the area. After hemostasis, the GelMA/MSN/HAp or GelMA/ICA@MSN/HAp scaffolds were implanted into the corresponding groups. The model and ICA gavage groups did not receive any scaffold implants. Two days after the surgery, the ICA gavage group received daily oral administration of ICA at a dose of 125 mg/kg for 8 weeks. Muscle and skin layers were sutured, and the wound was monitored for signs of bleeding. Postoperative care included daily intraperitoneal penicillin sodium injections for one week.

#### *3.3 Biocompatibility Evaluation*

To assess the in vivo safety of scaffold, biocompatibility evaluations were performed. At postoperative days 1, 7, 14 and 28, blood samples were collected from 6 rats per group. Serum levels of alanine aminotransferase (ALT), albumin (ALB), total protein (TP), aspartate aminotransferase (AST), γ-glutamyl transpeptidase (GGT), creatinine (Cre), blood urea nitrogen (urea), and uric acid (UA) were measured using a fully automated biochemical analyzer (Chemray 240, Wuhan Seville Technology).

#### *3.4 X-ray, Angiography, and Micro-CT Analysis*

Postoperative X-ray imaging was performed at 2, 4, 8, and 12 weeks using an X-ray machine (ZU-L5TYH, Hitachi Suzhou Medical Systems) to monitor the bone healing process dynamically. Lane-Sandhu X-ray scoring system was applied to evaluate bone formation, bone bridging, and bone remodeling at the femoral bone defect site after 12 weeks ^[1]^. Postoperative 12-week rats were euthanized, and Microfil angiography was performed. Briefly, after anesthesia, the thoracic cavity was opened to expose the aorta and the right atrium was cut. The vascular system was flushed with sterile saline containing 4 U/mL heparin sodium, followed by fixation with 4% paraformaldehyde. After removal of excess PFA with NaCl, Microfil was injected. The samples were stored overnight at 4 °C to allow the contrast agent to polymerize. The femora were dissected and fixed in 4% PFA for 48 h. Micro-CT imaging (Hiscan XM Micro CT, Suzhou Haesfeid Information Technology) was then performed to assess the trabecular thickness (Tb.Th), trabecular number (Tb.N) and bone volume/tissue volume (BV/TV) in the defect region. After decalcification with 10% EDTA (183004, Wuhan Kewi Biotech), blood vessel volume around the bone defect was visualized. The scanning parameters included a resolution of 9 μm, 80 kV, 100 µA, with a rotation angle of 0.6° per scan, totaling 180° per scan. Three rats per group were analyzed.

#### *3.5 Histological and Immunohistochemical Evaluation*

#### Liver and kidney tissues were collected at days 1, 7, 14, and 28 post-surgery, while femoral samples were harvested at 12 weeks. The collected tissues were dehydrated through graded ethanol and embedded in paraffin, followed by sectioning at 5 μm thickness. Coronal sections were prepared for liver and kidney tissues, whereas sagittal sections were obtained for bone samples. Hematoxylin and eosin (H&E) staining was performed for general histological assessment of liver, kidney, and bone tissues. Additionally, Masson’s trichrome staining was used to evaluate collagen deposition and fibrotic tissue formation, while Alizarin red staining was employed to assess calcium deposition in bone tissue. To further investigate osteogenesis and angiogenesis, immunohistochemical (IHC) staining was performed using primary antibodies against VEGF (bsm-52338R, 1: 100, Bioss), PROX-1 (bs-2774R, 1: 100, Bioss), EMCN (bs-5884R, 1: 100, Bioss), RUNX2 (bs-1134R, 1: 100, Bioss), COL1A1 (bs-0578R, 1: 100, Bioss), HIF-1α (GB151339-100, 1:600, Sevicebio), LDHA (bsm-62875R, 1:100, Bioss), PDK-1(bs-3788R, 1:100, Bioss). Tissue sections underwent deparaffinization, rehydration, and antigen retrieval, followed by overnight incubation at 4 °C with the respective primary antibodies. After washing, sections were incubated with horseradish peroxidase - conjugated secondary antibodies, and counterstained with hematoxylin. Finally, images were acquired and analyzed using Image-Pro Plus software (version 6.0, Media Cybernetics, USA).

#### *3.6 Immunofluorescence Analysis*

#### To evaluate the recruitment effect of the GelMA/ICA@MSN/HAp scaffold on LECs, HUVECs, and BMSCs, we compared the expression levels of LYVE-1, VEGFR3, and CD105 in the GelMA/MSN/HAp group and the GelMA/ICA@MSN/HAp group at both 4 and 8 weeks using immunofluorescence 3D reconstruction techniques. To evaluate the expression of BMP-2, VEGFR1 and PROX-1 in the bone formation region, triple immunofluorescence staining was performed. Tissue sections were incubated with primary antibodies against BMP-2 (bs-1012R, 1:100, Bioss), VEGF (bs-0170R, 1: 100, Bioss), and LYVE-1 (bs-2774R, 1: 100, Bioss) overnight at 4 °C. The next day, fluorescence-conjugated secondary antibodies were applied at room temperature for 1 h, followed by DAPI nuclear staining (0.5 µg/mL, 5 min at room temperature). The stained samples were then visualized using a Nikon ECLIPSE C1 inverted fluorescence microscope, and images were captured for analysis. The number of positive cells or fluore scently stained areas was quantified in five randomly selected fields across three slides per sample.

### *3.7 Transcriptome Sequencing and Analysis*

Total RNA was extracted from the tissue using TRIzol® Reagent following the manufacturer's instructions. RNA quality was assessed using the 5300 Bioanalyzer (Agilent) and quantified with the ND-2000 (NanoDrop Technologies). Only high-quality RNA samples were used for sequencing library construction. RNA purification, reverse transcription, library construction, and sequencing were conducted at Shanghai Majorbio Bio-pharm Biotechnology Co., Ltd. (Shanghai, China) following the manufacturer's instructions (Illumina, San Diego, CA). Differentially expressed genes (DEGs) between two different samples were identified by calculating the expression levels of each transcript according to the transcripts per million reads (TPM) method. Gene abundances were quantified using RSEM ^[2]^. Differential expression analysis was performed using DESeq2 or DEGseq ^[3]^. DEGs with |log2FC| ≥ 1 and FDR < 0.05 (DESeq2) or FDR < 0.001 (DEGseq) were considered significantly differentially expressed. Functional enrichment analysis, including Gene Ontology (GO) and Kyoto Encyclopedia of Genes and Genomes (KEGG) pathways, was performed to identify which DEGs were significantly enriched in GO terms and metabolic pathways. Enrichment was considered significant at a Bonferroni-corrected P-value < 0.05 relative to the entire transcriptome background. GO functional enrichment and KEGG pathway analysis were performed using Goatools and Python scipy software, respectively.

***4.Mechanism of activation of mitochondrial metabolic reprogramming during bone repair by GelMA/ICA@MSN/HAp composite scaffolds***

***4.1 Construction of co-culture system***

LECs were seeded in the upper chamber of the Transwell insert at a density of 5×10^4^ cells/cm³ and cultured in EGM-2 medium supplemented with 2% fetal bovine serum, growth factors, and scaffold extracts. After 24 h, once a confluent endothelial monolayer was formed, BMSCs and HUVECs were seeded into the lower chamber at densities of 2×10^4^ cells/cm^3^ and 3×10^4^ cells/cm³, respectively. BMSCs were cultured in α-MEM medium containing 10% FBS and scaffold extracts, while HUVECs were maintained in EGM-2 medium supplemented with 2% FBS, growth factors, and scaffold extracts. The HIF-1ɑ inhibitor PX-478 was used at a concentration of 20 μM. The co-culture system was then maintained for an additional 48 h to assess the effects of LECs on the proliferation and differentiation of BMSCs and HUVECs.Additionally, the content of CXCL12 in each group was analyzed using the ELISA assay kit.

***4.2 Assay of cellular respiration and glycolytic activity***

Cellular respiration and glycolytic activity were assessed by measuring the oxygen consumption rate (OCR) and extracellular acidification rate (ECAR) using a Seahorse XFe96 extracellular flux analyzer (Seahorse Bioscience, USA). BMSCs or HUVECs were seeded into Seahorse XF cell culture microplates and incubated in Seahorse Assay Medium. Prior to measurement, cells were incubated for 1 hour in a CO_2_-free environment to equilibrate. For the OCR assay, oligomycin, FCCP, and rotenone/antimycin A were sequentially injected to evaluate mitochondrial respiration. For the ECAR assay, glucose, oligomycin, and 2-deoxy-D-glucose (2-DG) were added sequentially to assess glycolytic function. The resulting OCR and ECAR values were recorded and analyzed to evaluate cellular metabolic activity.

### *4.3 Analysis of Single-Cell Sequencing and Spatial Transcriptomics Datasets*

### The single-cell RNA sequencing dataset (GSE192630) was analyzed to investigate bone tissue at three stages: before fracture, 5 days post-fracture, and 10 days post-fracture. Quality control was performed to ensure the reliability of the data. Cell populations were annotated using commonly expressed marker genes, including: immune cells (*Ptprc, Cd68, S100a8*), BMSCs (*Col1a1, Col1a2, Dcn*), cycling cells (*Mki67, Stmn1*), endothelial cells (*Plvap, Cdh5, Pecam1, Pdpn*), adipocytes (*Adipoq*), smooth muscle cells (*Acta2, Rgs5*), skeletal muscle cells (*Mylpf, Tnnc2*), and neurons (*Pou3f1*). Histograms were generated to visualize cell-type distributions. To investigate metabolic changes, expression levels of *Hif1α* and *Ldha* were analyzed in endothelial cells and BMSCs, comparing the “Control” and "Fracture" groups using t-tests. To further explore the spatial distribution of lymphatic endothelial cells during fracture healing, spatial transcriptomics (ST) data from the GSE203612 cohort were analyzed. These samples were obtained from mice that had undergone femoral osteotomy and were profiled using 10 × Genomics ST technology. Data processing and analysis were conducted using the Seurat R package (version 4.3.0). Raw counts were normalized, and spatial parameters were refined using the SCTransform function. Dimensionality reduction was performed using RunPCA and RunUMAP, and spatial expression patterns of key genes were visualized using SpatialFeaturePlot.

### *Statistical Analysis*

All bioinformatics statistical analyses were conducted using Graphpad Prism 9.5.0. Data were expressed as x ± s, and one-way ANOVA was used for comparisons among groups. Unless otherwise specified, all statistical tests were two-tailed, with *p* < 0.05 considered statistically significant, *p* < 0.01 as highly significant, and *p* < 0.001 as extremely significant.

**References**

[1] J. M. Lane, H. S. Sandhu, *Orthop Clin North Am* **1987**, *18*, 213.

[2] B. Li, C. N. Dewey, *BMC Bioinformatics* **2011**, *12*, 323.

[3] L. Wang, Z. Feng, X. Wang, X. Wang, X. Zhang, *Bioinformatics* **2010**, *26*, 136;

[4] M. I. Love, W. Huber, S. Anders, *Genome Biol* **2014**, *15*, 550.

**Table 1 Primer Sequences**

| **Primer** | **5’-3’ sequence** | |
| --- | --- | --- |
| ***Col1a1*** | **Forward:** | 5'-CCATTCGCAAACCAGTGTGG-3' |
|  | **Reverse:** | 5'-TGGGAGGGTTAAGCATCATACA-3' |
| ***Runx2*** | **Forward:** | 5'-ATCCAGCCACCTTCACTTACACC-3' |
|  | **Reverse:** | 5'-GGGACCATTGGGAACTGATAGG-3' |
| ***Vegf*** | **Forward:** | 5'-TACCTCGGGACTGGCTGTGG-3' |
|  | **Reverse:** | 5'-CCTGCGACCTGCTTCATTGTTG-3' |
| ***Prox-1*** | **Forward:** | 5'-ACGAGAAGGCAGCAACAAAGAAAG-3' |
|  | **Reverse:** | 5'-ACCTGAGGAACCTGGCGAGAG-3' |
| ***Emcn*** | **Forward:** | 5'-AGAACACAGTCTATGCCGTGGTG-3' |
|  | **Reverse:** | 5'-CCGCCTGTTGATTTGTTTGTGATTG-3' |
| ***Bmp-2*** | **Forward:** | 5'-AGAGTCAGATTACAGATCCCAGG-3' |
|  | **Reverse:** | 5'-TGGCTCTTCTTACTGAGAGAGG-3' |
| ***Pecam-1*** | **Forward:** | 5'-GGCACCATGAACAAACTAGCAAGG-3' |
|  | **Reverse:** | 5'-GGCAATGACCACTCCAATGACAAC-3' |
| ***Lyve-1*** | **Forward:** | 5'-CAAGTGTGGGAAGAATGGCAAAGG-3' |
|  | **Reverse:** | 5'-GAGGATGAAGCCGAGTAGGTGTC-3' |
| ***Podoplanin*** | **Forward:** | 5'-CCAGCCACTCCACGGACAAG-3' |
|  | **Reverse:** | 5'-GGGTCACTACAGCCAAGCCATC-3' |
| ***Hif-α*** | **Forward:** | 5'-TGATTTTACTCATCCGTGTGACC-3' |
|  | **Reverse:** | 5'-AAGCTCCTTTGCGTGTTTTGT-3' |
| ***Ldha*** | **Forward:** | 5′-GTTGTTGGGGTTGGTGCTGT-3′ |
|  | **Reverse:** | 5′-TCGCCCTTGAGTTTGTCTTC-3′ |
| ***Pdk-1*** | **Forward:** | 5′-GGACTTCGGGTCAGTGAATGC-3′ |
|  | **Reverse:** | 5′-TCCTGAGAAGATTGTCGGGGA-3′ |
| ***β-actin*** | **Forward:** | 5'-GATATCGCTGCGCTCGTC-3' |
|  | **Reverse:** | 5'-TGGGGTACTTCAGGGTCAGG-3' |
